# The metabolic influence of duodenal mucosal resurfacing for nonalcoholic fatty liver disease

**DOI:** 10.1097/MD.0000000000035147

**Published:** 2023-10-06

**Authors:** Te-Jung Chuang, Chung-Wang Ko, Sz-Iuan Shiu

**Affiliations:** a Division of Gastroenterology and Hepatology, Department of Internal Medicine, Tri Service General Hospital, Taipei, Taiwan; b Department of Internal Medicine, Taichung Veterans General Hospital, Taichung, Taiwan; c Division of Gastroenterology and Hepatology, Department of Internal Medicine, Taichung Veterans General Hospital, Taichung, Taiwan; d Department of Critical Care Medicine, Taichung Veterans General Hospital, Taichung, Taiwan.

**Keywords:** duodenal mucosal resurfacing, meta-analysis, nonalcoholic fatty liver disease, nonalcoholic steatohepatitis

## Abstract

**Background:**

Nonalcoholic fatty liver disease (NAFLD) or nonalcoholic steatohepatitis (NASH) is a leading cause of chronic liver disease worldwide with decreased life expectancy. Duodenal mucosal resurfacing (DMR) has been associated with metabolic improvement in glycemic and hepatic parameters of type 2 diabetes, but the metabolic impact of DMR for NAFLD/NASH remains inconclusive. We conducted a meta-analysis to investigate metabolic effects of DMR in patients with NAFLD/NASH.

**Methods:**

Three major bibliographic databases were reviewed for enrollment of trials prior to January 28, 2022. We included adults with biopsy-proven NAFLD/NASH or liver magnetic resonance imaging proton density fat fraction (MRI-PDFF) >5% at baseline and focused on the metabolic difference of MRI-PDFF at 12 weeks, and HbA1c or homeostatic model assessment index for insulin resistance (HOMA-IR) at 24 weeks.

**Results:**

Two studies involved a total of 67 participants for analysis. When compared with pre-intervention status, mean difference of MRI-PDFF, HbA1c, and HOMA-IR after DMR were −2.22 (95% CI: −12.79~8.34), −0.32% (95% CI: −0.80~0.16), and 0.15 (95% CI: −5.11~5.41) without statistical significance.

**Conclusions:**

For patients with NAFLD/NASH, DMR has the trend to improve liver fat at 12 weeks, and glycemic control in terms of HbA1c level at 24 weeks based on a very low quality of evidence.

## 1. Introduction

Nonalcoholic fatty liver disease (NAFLD) is hepatic steatosis in the absence of secondary causes of fat accumulation within liver^[1,2]^ and nonalcoholic steatohepatitis (NASH) involved liver parenchymal injury in the background of ≥5% hepatic steatosis, either by imaging or histology. NAFLD affects up to 25% of the population globally with nearly 60% of biopsied NAFLD presenting with NASH.^[3]^ NAFLD/NASH is not only a leading cause of chronic liver disease with estimated annual incidence of hepatocellular carcinoma ranging from 0.5% to 2.6% among patients progressed to cirrhosis^[4,5]^ but also associated with many metabolic diseases including obesity, hyperlipidemia, and type 2 diabetes mellitus (T2DM),^[6,7]^ which might increase overall and liver-specific mortality in patients with NASH and T2DM.^[8]^ Therefore, early diagnosis and intervention of NAFLD/NASH in clinical practice is an important issue to prevent disease progression.

Although variety of interventions has been advocated to treat patients with NAFLD/NASH including lifestyle modifications,^[9]^ nutritional supplementations,^[10]^ and pharmacologic therapies,^[11]^ there is still an unmet need for an effective treatment for NAFLD/NASH. Weight reduction surgery, also called bariatric surgery targeting the gastrointestinal tract, such as Roux-en-Y gastric bypass and sleeve gastrectomy, has been demonstrated to reverse steatosis, and decrease NAFLD activity scores based on a very low certainty of evidence in recent meta-analyses.^[12,13]^ But the surgical complications of bariatric surgery in obese cirrhotic patients was higher than that in patients without cirrhosis, and the mortality rates for compensated cirrhosis and decompensated cirrhosis were 0.9% and 18.2%, respectively.^[14]^ Another meta-analysis also reported that the overall complications, postoperative bleeding, length of hospital stay, and in-hospital/90-day mortality were significantly higher in patients with compensated cirrhosis than that without cirrhosis.^[15]^ As a result, only minority of the eligible obesity population received surgical interventions, leaving the majority of patients undertreated.^[16]^ Recently, endoscopic bariatric and metabolic therapies (EBMTs) have been introduced for NAFLD/NASH in a minimally invasive and cost-effective manner and served as an alternative modality for patients who are contraindicated or unwilling to undergo bariatric surgery.^[17]^ EBMTs involved intragastric balloons (IGBs), endoscopic sleeve gastroplasty (ESG), duodenal-jejunal bypass liner (DJBL), and duodenal mucosal resurfacing (DMR), which is a novel endoscopic procedure involving catheter-based hydrothermal ablation of duodenal mucosa followed by subsequent regeneration^[18]^ and aims to treat metabolic disorders by decreasing anti-incretins’ role and reestablishing a healthy neuroendocrine axis with new enterocytes formation. In the latest REVITA-2 trial^[19]^ in patients with T2DM, DMR might exert beneficial metabolic effects when compared to sham procedure, but there were few literatures investigating the metabolic effects of DMR in patients with NAFLD/NASH, leaving the clinical impact of DMR in this group inconclusive. Therefore, the aim of our systematic review and meta-analysis was to investigate metabolic effects of DMR in patients with NAFLD/NASH.

## 2. Materials and methods

### 2.1. Search strategy and selection criteria

This systematic review was performed according to Preferred Reporting Items for Systematic Reviews and Meta-Analyses 2020 recommendations.^[20]^ Comprehensively computerized research was performed on the electronic databases including PubMed, Embase, and the Cochrane Central Register of Controlled Trials prior to January 28, 2022, without any language restrictions. Additionally, we conducted a manual search of references in retrieved articles and relevant reviews for eligible publications and registered the protocol in PROSPERO (CRD 42022349101). A detailed description of the search strategies is provided in Table S1 (see Table 1, Supplemental Content, http://links.lww.com/MD/K8, which illustrates the searching strategy of electronic database). Ethical approval and informed consent from the participants were not necessary as there was no individual participant data involved.

We included trials that assessed the efficacy of DMR in adults with biopsy-proven NAFLD/NASH or liver magnetic resonance imaging proton density fat fraction (MRI-PDFF) >5% at baseline. Studies with concern for duplication in the population of patients, patients on concomitant pharmacological interventions, or additional procedures were excluded.

### 2.2. Outcome measures

We determined the clinical response of MRI-PDFF at 12 weeks, and HbA1c or homeostatic model assessment index for insulin resistance (HOMA-IR) at 24 weeks after completion of the therapeutic intervention from enrolled studies.

### 2.3. Data extraction and quality assessment

Two investigators (C-TJ and K-CW) screened the titles and abstracts for eligibility independently and evaluated full texts in order to clarify the eligibility status accordingly. Any disagreements were discussed until a consensus was reached, with a third investigator (S-SI) being consulted when necessary. One reviewer (C-TJ) extracted data, which was checked by a second investigator (S-SI) subsequently. We attempted to contact the corresponding authors if relevant outcomes were not indicated in the article. The following variables were extracted: study design, participants’ characteristics, detailed interventions, and outcome measurements. Two authors (C-TJ and S-SI) evaluated the risk of bias based upon the version 2 of the Cochrane tool for assessing Risk of Bias in randomized trials (RoB 2.0 tool)^[21,22]^ for randomized trials and the Risk Of Bias In Non-randomized Studies – of Interventions (ROBINS-I tool) for non-randomized studies.^[23]^ Disagreements were discussed until a consensus was reached.

### 2.4. Data synthesis and statistical analysis

The results were analyzed by Review Manager V.5.3 software (Nordic Cochrane Centre, Copenhagen, Denmark). The pooled mean difference (MD) and 95% confidence interval (CI) were reported for dichotomous variables of the short-term outcomes of metabolic effects in patients with NAFLD/NASH undergone DMR. These were completely produced using a random effect model to allow for the expected heterogeneity amongst the enrolled studies.

Heterogeneity of the outcome measures was examined using the Cochrane *I*^2^ statistic. We regarded an *I*^2^ <25% as mild heterogeneity, 25%–50% as moderate heterogeneity, and higher than 50% as severe heterogeneity. If the *x*^2^ test showed *P* > .05 it was not considered significant in the heterogeneity test of the research. We checked for publication bias by carrying out visual inspection of the funnel plot.

## 3. Results

**Figure 1.**
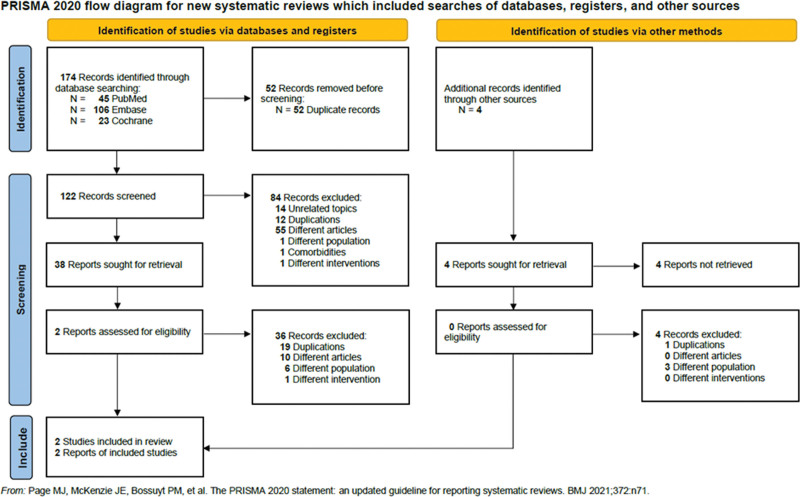
PRISMA 2020 flow diagram. PRISMA = preferred reporting items for systematic reviews and meta-analyses.

The detailed searching strategy is summarized in Figure 1. Initially, we screened 122 abstracts and reviewed 38 full-text articles independently after exclusion of 84 studies which were not relevant to our topic. Finally, we included 2 articles involving a total of 67 participants for the purpose of qualitative and quantitative synthesis.

### 3.1. Characteristics of the included studies

The methodology and characteristics of study design and outcomes of 1 randomized controlled trial and 1 prospective cohort study, are summarized in Table S2 (see Table 2, Supplemental Content, http://links.lww.com/MD/K9, which demonstrates the methodology of enrolled trials), Table S3 (see Table 3, Supplemental Content, http://links.lww.com/MD/K10, which demonstrates the technical characteristics of enrolled trials), and Table S4 (see Table 4, Supplemental Content, http://links.lww.com/MD/K11, which demonstrates the outcome characteristics of enrolled trials). Amongst these studies, the sample size ranged from 11 to 108, while the ranges for age and percentage of male gender were 50.0 to 58.2, and 18.0 to 69.4, respectively. These studies were conducted in the Europe and Brazil. The percentage of participants with diabetes ranged from 82.0 to 100.0 while the percentage for body mass index (BMI) was 31.5 to 32.1. The percentage of serious adverse events after intervention was 3.6 to 18.2%.

### 3.2. Outcome parameters

**Figure 2.**
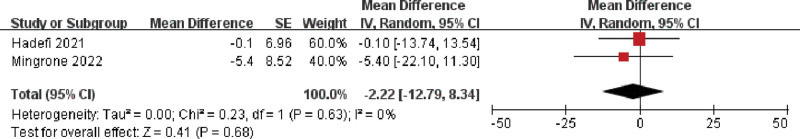
Forest plot of pooled mean difference of MRI-PDFF 12–24 wk after receiving duodenal mucosal resurfacing. CI = confidence interval, MRI-PDFF = magnetic resonance imaging proton density fat fraction, SE = standard error.

**Figure 3.**
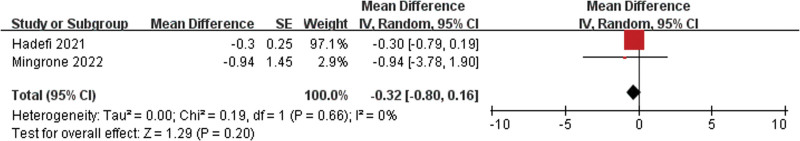
Forest plot of pooled mean difference of HbA1c 24 wk after receiving duodenal mucosal resurfacing. CI = confidence interval, SE = standard error.

**Figure 4.**
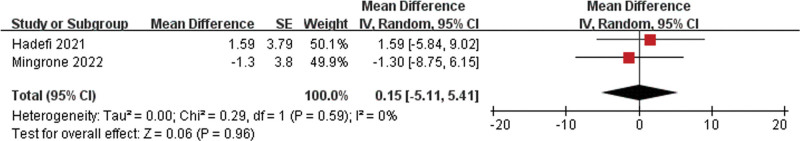
Forest plot of pooled mean difference of HOMA-IR 24 wk after receiving duodenal mucosal resurfacing. CI = confidence interval, HOMA-IR = homeostatic model assessment index for insulin resistance, SE = standard error.

The pooled MD of MRI-PDFF at 12 weeks after receiving DMR was −0.22 (95% CI, −12.79~8.34, *P* = .63, Fig. 2), while the pooled MD of HbA1c at 24 weeks, and HOMA-IR at 24 weeks were −0.32 (95% CI, −0.80~0.16, *P* = .66, Fig. 3), and 0.15 (95% CI, −5.11~5.41, *P* = .59, Fig. 4) respectively.

The Cochrane Collaboration Risk of Bias assessment is shown in Table S5 (see Table 5, Supplemental Content, http://links.lww.com/MD/K12, which illustrates the risk of bias for randomized trials). Some concern was existing in overall bias because these studies did not describe the randomization process completely. ROBINS-I assessment is shown in Table S6 (see Table 6, Supplemental Content, http://links.lww.com/MD/K13, which illustrates the risk of bias in non-randomized studies), which showed that critical risks of bias was distributed in the domains of bias due to confounding resulting in a critical risk of overall bias. Mild to moderate risks of bias were distributed in the domains of selection bias, bias in classification of interventions, deviation from intended interventions, and bias due to missing data attrition.

## 4. Discussion

In this meta-analysis, we comprehensively investigated metabolic effects of DMR in patients with NAFLD/NASH, and reported that pooled MD of MRI-PDFF, HbA1c, and HOMA-IR after DMR were −2.22 (95% CI: −12.79~8.34), −0.32% (95% CI: −0.80~0.16), and 0.15 (95% CI: −5.11~5.41) without statistical significance when compared with pre-intervention status. For patients with NAFLD/NASH, DMR might be predisposed to improve liver fat at 12 weeks, and HbA1c level at 24 weeks except HOMA-IR.

In recent years, EBMTs have emerged as an alternative modality of bariatric surgery for patients who are not permitted or unwilling to receive general anesthesia^[24]^ and demonstrated a significant impact on body weight loss and metabolic parameters with reasonable safety in literature review.^[25]^ Although there was no head-to-head comparison between EBMTs and bariatric surgery at present, BMI loss of EBMTs are generally lower than that of bariatric surgery^[25,26]^ with fewer complication rates of EBMTs when compared to bariatric surgery. EBMTs are usually divided into gastric and small bowel categories according to instrument design and mechanisms of actions. IGBs functioned by the temporary inflation of a balloon into the stomach to achieve delayed gastric emptying^[27,28]^ while ESG utilized endoscopic suturing device to modify the gastric structure to reduce accommodation and gastric volume, both of which eventually resulted in reduced caloric consumption. One recent meta-analysis^[25]^ reported that IGB achieved a decreasing percentage of total body weight loss at 6 months significantly when compared to the standard medication with weighted MD of 5.45 (95% CI: 3.88~7.05) and reported slightly lower percentage of total body weight loss at 12 months between 2 groups. Only 1 pilot study conducted by Lee YM et al^[29]^ found that NAFLD activity scores was better in the IGB group with statistical significance while there was no difference in histological findings between both groups, which was inconsistent with another prospective study, which demonstrated that both metabolic and histologic improvements was facilitated by IGB significantly.^[30]^ As for ESG, 2 clinical trials^[31,32]^ found statistically significant body weight loss when compared to lifestyle modifications, and another 2 studies^[33,34]^ indicated clinical improvement of hepatic steatosis and fibrosis in patients with NAFLD as well as metabolic parameters. The adverse events of IGBs are generally mild with one-fifth of them having premature removal due to intolerance and complications while serious events of ESG were estimated about 5% in literature review.^[25]^ In our review, serious adverse events after DMR intervention varied widely from 3.6 to 18.2% which was higher when compared to IGBs and ESG indirectly.

As for the small bowel counterpart of EBMTs, DJBL anchored at the duodenal bulb with flexible sleeve made from Teflon, which covered the enteric mucosal surface till the proximal jejunum, to bypass the biliopancreatic limb and limit nutrients contact to the mucosa. Lee YM et al^[25]^ showed that DJBL have no significant reduction in BMI but improved HbA1c at 12 months when compared with standard medication group. Another retrospective study^[35]^ including diabetic patients after DJBL 1 year later had shown a significant reduction of liver fibrosis. However, 1 systemic review^[36]^ revealed that severe adverse events was 3.7% associated with DJBL with higher rate of hepatic abscess than global incidence, and Caiazzo R et al^[37]^ observed severe complications in nearly 40% of enrolled participants which resulted in unanticipated removal of the device in one-seventh of them, which indicated the modification of the anchoring device is warranted to prevent early migration, occlusion, gastrointestinal bleeding, or perforation.

Insulin resistance^[38]^ is the pathophysiologic etiology of several metabolic disorders including diabetes and fatty liver disease, and nutrient delivery to jejunum without contact to duodenum demonstrated to increase the insulin sensitivity in human subjects.^[39]^ DMR restores the normal mucosal interface by resurfacing via hydrothermal ablation based on the assumption that the duodenal surface is mediating an abnormal signal damaging the endogenous insulin-sensitive tissues. In a multi-centered study,^[40]^ DMR elicited glycemic improvement in type 2 diabetes patients who already took oral glucose-lowering medications irrespective of weight loss. Another study demonstrated the potential of discontinuing insulin therapy when DMR and glucagon-like peptide-1 receptor agonist were administered simultaneously to patients with type 2 diabetes, who were already using insulin.^[41]^ Two meta-analyses^[25,42]^ regarding the metabolic influences of endoscopic DMR have been published but de Oliveira GHP et al enrolled duplicated poster^[43]^ from REVITA-2 trial^[19]^ while the other included a trial focusing on insulin resistant women with polycystic ovary syndrome^[44]^ rendering the selection bias in their interpretation. The European cohort of REVITA-2 trial achieved better HbA1c and liver-fat reduction after DMR than sham group, and we also demonstrated that DMR has the trend to improve liver fat at 12 weeks, and HbA1c level at 24 weeks after DMR, which indicated that the difference of liver fat might be correlated with improvement of glycemic control.

There are several limitations to this meta-analysis. First of all, we enrolled 1 randomized controlled trial and 1 open-labeled pilot study in our analysis, which contributed to selection bias and publication bias. We excluded most of the studies because of noncorrelation between DMR and NAFLD/NASH. Concerning the novelty of the DMR procedure, we enrolled a small sample size involving 67 participants within the setting of highly heterogeneous disease of NAFLD/NASH, which might devote to confounding factors and outcome differences. In REVITA-2 trial, there were also some differences in baseline insulin concentrations, numbers of medications, and disease duration of diabetes between the European and Brazilian cohorts, which also resulted in the regional differences in subgroup outcomes. On the contrary, we did not perform subgroup analysis owing to small sample size and sparse number of enrolled trials. Secondly, it is challenging to estimate the clinical outcomes of NAFLD/NASH after DMR procedure when reporting bias exists due to discrete outcome definitions, assessments, and follow up duration as well as shortage of head-to-head comparisons for different types of modalities in the treatment of NAFLD/NASH. Therefore, further multi-center prospective studies are awaited, particularly in different interventional comparisons of EBMTs and bariatric surgery. Thirdly, variability in outcome measurement assaulted clinical outcome of steatohepatitis and liver fibrosis, which predisposed of measurement bias in our meta-analysis. One latest diagnostic meta-analysis^[45]^ investigated the diagnostic accuracy of NASH in 4 studies using magnetic resonance elastography and showed that the sensitivity and specificity were 65% and 83%, respectively. Further intention-to-diagnose analyses are needed as well as external validation of potential index tests when compared to liver biopsy.

## 5. Conclusions

In this meta-analysis focusing on NASH/NAFLD, DMR has the trend to improve liver fat at 12 weeks with better glycemic control in terms of HbA1c level at 24 weeks based on a very low quality of evidence. Further studies to confirm the efficacy and safety of DMR and elucidate the metabolic mechanisms are assured for patients with fatty liver disease.

## Acknowledgments

The authors acknowledge the contribution from the Evidence-based Practice and Policymaking Committee, Taichung Veterans General Hospital, Taichung, Taiwan.

## Author contributions

**Conceptualization:** Te-Jung Chuang.

**Data curation:** Te-Jung Chuang.

**Formal analysis:** Chung-Wang Ko, Sz-Iuan Shiu.

**Project administration:** Te-Jung Chuang.

**Supervision:** Chung-Wang Ko, Sz-Iuan Shiu.

**Writing – original draft:** Te-Jung Chuang.

**Writing – review & editing:** Sz-Iuan Shiu.

## Supplementary Material
