# Supplementary material for: The metabolic influence of duodenal mucosal resurfacing for nonalcoholic fatty liver disease
Source: Medicine (Baltimore). 2023 Oct 6;102(40):e35147. doi: 10.1097/MD.0000000000035147 (PMC10553053; doi:10.1097/MD.0000000000035147)
Supplement: Supplementary file 1 [file medi-102-e35147-s001.doc]

**Supplementary Table 1. Electronic Database Searching Strategy**

| **PubMed searching strategy** | | |
| --- | --- | --- |
| Intervention | #1 | (((Mucosa[Title/Abstract] OR Mucosal[Title/Abstract]) AND (Resurface[Title/Abstract] OR Resurfacing[Title/Abstract])) OR (Hydrothermal[Title/Abstract] AND (Ablation[Title/Abstract] OR Ablating[Title/Abstract]))) AND (Duodenum[Title/Abstract] OR Duodenal[Title/Abstract]) |
| **Embase searching strategy** | | |
| Intervention | #1 | (((Mucosa:ti,ab,kw OR Mucosal:ti,ab,kw) AND (Resurface:ti,ab,kw OR Resurfacing:ti,ab,kw)) OR (Hydrothermal:ti,ab,kw AND (Ablation:ti,ab,kw OR Ablating:ti,ab,kw))) AND (Duodenum:ti,ab,kw OR Duodenal:ti,ab,kw) |
| **Cochrane clinical trial search strategy** | | |
| Intervention | #1 | (((Mucosa:ti,ab,kw OR Mucosal:ti,ab,kw) AND (Resurface:ti,ab,kw OR Resurfacing:ti,ab,kw)) OR (Hydrothermal:ti,ab,kw AND (Ablation:ti,ab,kw OR Ablating:ti,ab,kw))) AND (Duodenum:ti,ab,kw OR Duodenal:ti,ab,kw) |
