# Supplementary material for: The metabolic influence of duodenal mucosal resurfacing for nonalcoholic fatty liver disease
Source: Medicine (Baltimore). 2023 Oct 6;102(40):e35147. doi: 10.1097/MD.0000000000035147 (PMC10553053; doi:10.1097/MD.0000000000035147)
Supplement: Supplementary file 2 [file medi-102-e35147-s002.doc]

**Supplementary Table 2. Narration of Enrolled Trials**

| **First author** | **Year** | **Country (No. of centers)** | **Sample size** | **Comparison intervention** | **Inclusion criteria** | **Outcome measures** |
| --- | --- | --- | --- | --- | --- | --- |
| **Hadefi A, et al.^2^** | 2021 | Belgium (1) | 11 | DMR (11) | Definite biopsy-proven NASH | - Δ Liver enzymes (AST, ALT) at 24 weeks  - Δ Fib-4/NAFLD fibrosis score at 24 weeks  - Δ MRI-PDFF at 24 weeks  - Liver histology (improvement of fibrosis with no worsening of NASH or NASH resolution with no worsening of fibrosis) at 12 months  - Δ HbA1c at 24 weeks  - Δ HOMA-IR at 24 weeks  - Adverse events |
| **Mingrone G, et al.^3^** | 2022 | Europe (9), Brazil (2) | 108 | DMR (56) *vs* Sham procedure (52) | Type II DM with preserved insulin secretion +  24 ≤ BMI ≤40 kg/m2 +  Currently taking ≥ 1 oral glucose-lowering medication | - Absolute ΔMRI-PDFF at 12 weeks  - Relative ΔMRI-PDFF at 12 weeks  - Δ HbA1c at 24 weeks  - Δ HOMA-IR at 24 weeks  - Adverse events |

*vs*, versus; DMR, duodenal mucosal resurfacing; NA, not available; NASH, nonalcoholic Steatohepatitis; DM, diabetes mellitus; BMI, body mass index; AST, aspartate aminotransferase; ALT, alanine aminotransferase; Fib-4, fibrosis-4; NAFLD, nonalcoholic fatty liver disease; MRI-PDFF, magnetic resonance imaging proton density fat fraction; HbA1c, glycated hemoglobin; HOMA-IR, homeostatic model assessment index for insulin resistance; TG, triglycerides; LDL, low-density lipoprotein; HDL, high-density lipoprotein.
