# Supplementary material for: The metabolic influence of duodenal mucosal resurfacing for nonalcoholic fatty liver disease
Source: Medicine (Baltimore). 2023 Oct 6;102(40):e35147. doi: 10.1097/MD.0000000000035147 (PMC10553053; doi:10.1097/MD.0000000000035147)
Supplement: Supplementary file 3 [file medi-102-e35147-s003.doc]

**Supplementary Table 3. Technical** Characteristics of Enrolled Trials

| **First author** | **Intervention groups** | **Intervention group 1** | **Intervention group 2** | **Postoperative care** |
| --- | --- | --- | --- | --- |
| **Hadefi A, et al.** | DMR (11) | NA | None | 2-week diet in which clear liquids were progressively replaced by solid food + Proton pump inhibitors (40 mg once a day) for 1 month + no dietary counseling |
| **Mingrone G, et al.** | DMR (56) *vs* Sham procedure (52) | Five sequential ablations of 2 axial centimetres each, starting within 3 centimetres distal to the Ampulla of Vater towards the Ligament of Treitz, totaling 10 axial centimetres of circumferentially ablated tissue in the duodenum | The sham procedure consisted of placing the DMR catheter over the guidewire into the stomach and leaving it in place for 30 min prior to removing it from the patient | NA |

DMR, duodenal mucosal resurfacing; NA, not available.
