# Supplementary material for: The metabolic influence of duodenal mucosal resurfacing for nonalcoholic fatty liver disease
Source: Medicine (Baltimore). 2023 Oct 6;102(40):e35147. doi: 10.1097/MD.0000000000035147 (PMC10553053; doi:10.1097/MD.0000000000035147)
Supplement: Supplementary file 4 [file medi-102-e35147-s004.doc]

**Supplementary Table 4. Outcomes Characteristics of Enrolled Trials**

| **First author** | **Mean age (years)** | **Male (%)** | **DM (%)** | **BMI**  **(kg/m^2^)** | **Intervention group** | **Outcome measures** | **Pre-intervention** | **Post-intervention** |
| --- | --- | --- | --- | --- | --- | --- | --- | --- |
|  |  |  |  |  |  |  | Event/Total | ITT (%) |
| **Hadefi A, et al.** | 50.0 | 18 | 82.0 | 32.1 | DMR (11) | 12 months | 3/11 | 27.2 |
|  |  |  |  |  |  | Improvement of fibrosis |  |  |
|  |  |  |  |  |  | Resolution of NASH | 0/11 | 0 |
|  |  |  |  |  |  | Serious adverse events | 2/11 | 18.2 |
|  |  |  |  |  |  | 24 weeks | 54 U/L (39 - 90) | 47 U/L (36 - 71) |
|  |  |  |  |  |  | Liver enzymes (ALT) (median [IQR]) |  |  |
|  |  |  |  |  |  | Fib-4 score (median [IQR]) | 1.36 (1.06 - 1.54) | 1.20 (0.98 - 1.34) |
|  |  |  |  |  |  | NAFLD fibrosis score (median [IQR]) | -1.14 (-1.89 - -0.32) | -0.95 (-1.58 - -0.46) |
|  |  |  |  |  |  | MRI-PDFF (median [IQR]) | 23% (16 - 28) | 22% (19 – 26) |
|  |  |  |  |  |  | HbA1c (median [IQR]) | 6.5% (6.35 - 6.75) | 6.2% (6.10 - 6.40) |
|  |  |  |  |  |  | HOMA-IR (median [IQR]) | 6.3 (4.5 - 8.2) | 8.3 (4.5 - 11.1) |
| **Mingrone G, et al.** | 58.2 | 69.4 | 100.0 | 31.5 | DMR (56) *vs* Sham procedure (52) | Serious adverse events | 2/56 | 3.6 |
|  |  |  |  |  |  | 12 weeks | -5.4 (5.6) | |
|  |  |  |  |  |  | Δ MRI-PDFF (median [IQR]) |  |  |
|  |  |  |  |  |  | 24 weeks | 65.6 mmol/mol (8.7) | 55.2 mmol/mol (16.4) |
|  |  |  |  |  |  | HbA1c (median [IQR]) |  |  |
|  |  |  |  |  |  | Δ HOMA-IR (median [IQR]) | -1.3 (2.5) | |

DM, diabetes mellitus; BMI, body mass index; ITT, intention-to-treat; DMR, duodenal mucosal resurfacing; NASH, nonalcoholic Steatohepatitis; ALT, alanine aminotransferase; IQR, interquartile range; Fib-4, fibrosis-4; NAFLD, nonalcoholic fatty liver disease; MRI-PDFF, magnetic resonance imaging proton density fat fraction; HbA1c, glycated hemoglobin; HOMA-IR, homeostatic model assessment index for insulin resistance.
