# Supplementary material for: The metabolic influence of duodenal mucosal resurfacing for nonalcoholic fatty liver disease
Source: Medicine (Baltimore). 2023 Oct 6;102(40):e35147. doi: 10.1097/MD.0000000000035147 (PMC10553053; doi:10.1097/MD.0000000000035147)
Supplement: Supplementary file 5 [file medi-102-e35147-s005.doc]

**Supplementary Table 5. Risk of Bias for Randomised Trials Involving Duodenal Mucosal Resurfacing**

| **Author** | **Year** | **Randomization process** | **Deviations from intended interventions** | **Missing outcome data** | **Measurement of the outcome** | **Selection of the reported result** | **Overall Bias** |
| --- | --- | --- | --- | --- | --- | --- | --- |
| **Mingrone G, et al.** | **2022** | Some concerns | Low risk | Low risk | Low risk | Low risk | Some concerns |
| NI, no information. |  | | | | | | |
