# Supplementary material for: The metabolic influence of duodenal mucosal resurfacing for nonalcoholic fatty liver disease
Source: Medicine (Baltimore). 2023 Oct 6;102(40):e35147. doi: 10.1097/MD.0000000000035147 (PMC10553053; doi:10.1097/MD.0000000000035147)
Supplement: Supplementary file 6 [file medi-102-e35147-s006.doc]

**Supplementary** **Table 6. Risk of Bias In Non-randomised Studies - of Interventions for Non-randomised Trials of Duodenal Mucosal Resurfacing**

| **Author** | **Year** | **Pre-intervention** | | **At-intervention** | **Post-intervention** | | | |
| --- | --- | --- | --- | --- | --- | --- | --- | --- |
|  |  | **Bias due to confounding** | **Selection bias** | **Bias in classification of interventions** | **Deviation from intended interventions** | **Bias due to missing data attrition** | **Bias in measurement of outcomes** | **Bias in selection of reported results** |
| **Hadefi A, et al.** | **2021** | Critical | Moderate | Moderate | Moderate | Moderate | Low | Low |
